# Systemic Adverse Events after Intravitreal Bevacizumab versus Ranibizumab for Age-Related Macular Degeneration: A Meta-Analysis

**DOI:** 10.1371/journal.pone.0109744

**Published:** 2014-10-16

**Authors:** Wei Wang, Xiulan Zhang

**Affiliations:** Zhongshan Ophthalmic Center, State Key Laboratory of Ophthalmology, Sun Yat-Sen University, Guangzhou, People's Republic of China; Medical University Graz, Austria

## Abstract

**Objective:**

To assess whether the incidence of systemic adverse events differs between those who used bevacizumab and those who used ranibizumab in the treatment of age-related macular degeneration (AMD).

**Methods:**

A systematic literature search was conducted to identify randomised controlled trials (RCTs) comparing the use of intravitreal bevacizumab with the use of ranibizumab in AMD patients. Results were expressed as risk ratios (RRs) with accompanying 95% confidence intervals (CIs). The data were pooled using the fixed-effect or random-effect model according to the heterogeneity present.

**Results:**

Four RCTs were included in the final meta-analysis. Overall, the quality of the evidence was high. There were 2,613 treated patients: 1,291 treated with bevacizumab and 1,322 treated with ranibicizumab. No significant differences between bevacizumab use and ranizumab use were found in terms of the incidence of death from all causes, arteriothrombotic events, stroke, nonfatal myocardial infarction, vascular death, venous thrombotic events, and hypertension, with the pooled RRs being 1.11 (0.77, 1.61), 1.03 (0.69,1.55), 0.84 (0.39,1.80), 0.97 (0.48, 1.96), 1.24 (0.63, 2.44), 2.38 (0.94, 6.04), and 1.02 (0.29, 3.62), respectively.

**Conclusions:**

The meta-analysis shows that both treatments are comparably safe. However, the findings from our study must be confirmed in future research via well-designed cohort or intervention studies because of the limited number of studies.

## Introduction

Age-related macular degeneration (AMD) is the most common cause of blindness in individuals over 50 years of age [1]–[3]. Although an estimated 80% of patients with AMD have the non-neovascular (dry) form, the neovascular (wet) form is responsible for almost 90% of severe visual losses resulting from AMD [4]–[6]. Vascular endothelial growth factor-A (VEGF-A) has been proven to play a major role in the pathogenesis of wet AMD [7], [8]. Since the mid-2000s, antivascular endothelial growth factor (anti-VEGF) therapy has become the mainstay of treatment for wet AMD [9].

Ranibizumab (Lucentis, Genentech, Inc., South San Francisco, CA, USA) is a recombinant humanized immunoglobulin G1κ isotype monoclonal antibody fragment directed toward all isoforms of VEGF-A [7]. It has been approved for the treatment of wet AMD by the food and drug administration (FDA) in the US (2006), Europe (2007), Japan (2009), and many other countries. However, the cost of ranibizumab is immense: monthly injections at a dose of 0.5 mg result in an annual cost greater than US $23,000 per patient [10].

Similar to ranibizumab, bevacizumab (Avastin, Genentech, Inc., South San Francisco, CA, USA) is a recombinant humanized full-length antibody that can inhibit all isoforms of VEGF-A [11]. In 2004, it was approved for the treatment of metastatic cancer of the colon or rectum, but it has not gained FDA approval for intravitreal use. Therefore, it can be utilized only in an off-label setting. For the past several years, it has been used off-label to treat wet AMD with very encouraging results. Bevacizumab has attracted more and more interest because of its low cost, which is especially important considering the number of injections that are necessary at 4- to 6-week intervals. A report suggested that the US medicare system could save more than US$1 billion within 2 years if bevacizumab replaced ranibizumab [7], [10].

Although anti-VEGF agents are injected in small quantities into the eye, concerns about systemic safety have been raised, especially for the off-label use of bevacizumab. Research has shown that the systemic administration of bevacizumab, along with chemotherapeutic agents, can increase the risk of thromboembolic events two-fold over chemotherapy alone [12]. Many recently published randomized clinical trials (RCTs) have evaluated intravitreal bevacizumab and ranibizumab for the treatment of wet AMD. The results of the comparison of the AMD Treatments Trial (CATT) and the Age-related Choroidal Neovascularization Trial (IVAN) demonstrated that bevacizumab was not inferior to ranibizumab in the treatment of wet AMD [13], [14]. However, these studies were not sufficiently powerful to detect drug-specific differences in the rates of systemic adverse events. Hence, the crucial question of whether adverse effects differ between off-label bevacizumab and licensed ranibizumab has not yet been answered [15].

To determine whether the intravitreal injection of bevacizumab creates a higher risk of systemic adverse events than ranibizumab injection does, we undertook a systematic review and meta-analysis of all relevant head-to-head RCTs.

## Methods

This study was reported in accordance with the Preferred Reporting Items for Systematic Reviews and Meta-Analyses (PRISMA) statement (Checklist S1) [16]. All stages of study selection, data extraction, and quality assessment were performed independently by two reviewers (W.W. and X.Z.). Any disagreement was resolved via discussion and consensus.

### 1. Literature Search

Studies were identified through a systematic search of Pubmed, Embase, the Chinese Biomedicine Database, and the Cochrane library from inception up to December 2013. The initial search terms were (Ranibizumab or Lucentis) AND (Bevacizumab or Avastin) AND (“Macular degeneration” or AMD), which were filtered by “Humans” and “Randomized Controlled Trial.” In addition, the reference lists of identified studies were manually checked to include other potentially eligible trials. This process was performed iteratively until no additional articles could be identified.

### 2. Study Selection

Studies were considered acceptable for inclusion in the meta-analysis if they met the following criteria: (1) the study design included randomized clinical trials; (2) the population was patients with wet AMD; (3) the interventions were intravitreal bevacizumab and intravitreal ranibizumab, which were directly compared in head-to-head design; (4) the incidence of systemic adverse events was reported; (5) there was a follow-up time of at least 1 year; and (6) there were at least ten patients in each arm. If there were multiple reports for a particular study, the most recent publication was included. Trials were excluded if they (1) were abstracts, letters, or meeting proceedings; (2) had repeated data or did not report outcomes of interest; or (3) included patients with other indications than wet AMD, patients previously treated with VEGF inhibitors, or patients receiving systemic anti-VEGF therapy.

### 3. Data Extraction

The following information was extracted from each study: first author; year of publication; study design; inclusion and exclusion criteria; number of patients in each group; characteristics of the study population; adverse events; the period, and number of injections preceding an adverse event. A Thromboembolic Event (TEE) was defined as any arteriothrombotic or venous thrombotic event [17].

### 4. Quality Assessment

The methodological quality of each trial was evaluated using the Jadad scale [18]. The scale consists of three items describing randomization (0–2 points), blinding (0–2 points), and dropouts and withdrawals (0–1 points) in the reporting of a randomized controlled trial. A score of 1 is given for each of the points described. A further point is awarded when the method of randomization and/or blinding is given and is appropriate, whereas when it is inappropriate, a point is deducted. The quality scale ranges from 0 to 5 points. Higher scores indicate better reporting. The studies are said to be of low quality if the Jadad score is ≤2 and of high quality if the score is ≥3.

### 5. Statistical Analysis

All outcomes were expressed as risk ratios (RRs) with accompanying 95% confidence intervals (CIs). Outcome measure was assessed on an intent-to-treat (ITT) basis, the ITT population being comprised of all randomized patients who received the study medication and provided a valid baseline measurement. The cochrane Q test was used to detect the heterogeneity of the effects. Significant heterogeneity was defined as a P value of <0.05. A fixed-effects model or random-effects model was used, depending on the presence or absence of heterogeneity. The I*^2^* value was used to demonstrate the percentage of the variability attributable to heterogeneity rather than to sampling error. Studies with an I*^2^* statistic of <25% are considered to have no heterogeneity, those with an I*^2^* statistic of 25% to 50% are considered to have low heterogeneity, those with an I*^2^* statistic of 50% to 75% are considered to have moderate heterogeneity, and those with an I*^2^* statistic of >75% are considered to have high heterogeneity [19]. Sensitivity analyses were performed by investigating the influence of a single study on the overall pooled estimate via omitting one study at a time. Potential publication bias was assessed by using Begg's and Egger's tests [20], [21]. A P value <0.05 was judged to be statistically significant, except when otherwise specified. All statistical analyses were performed using Stata version 12.0 (Stata Corp, College Station, TX).

## Results

### 1. Literature Search

**Figure 1:**
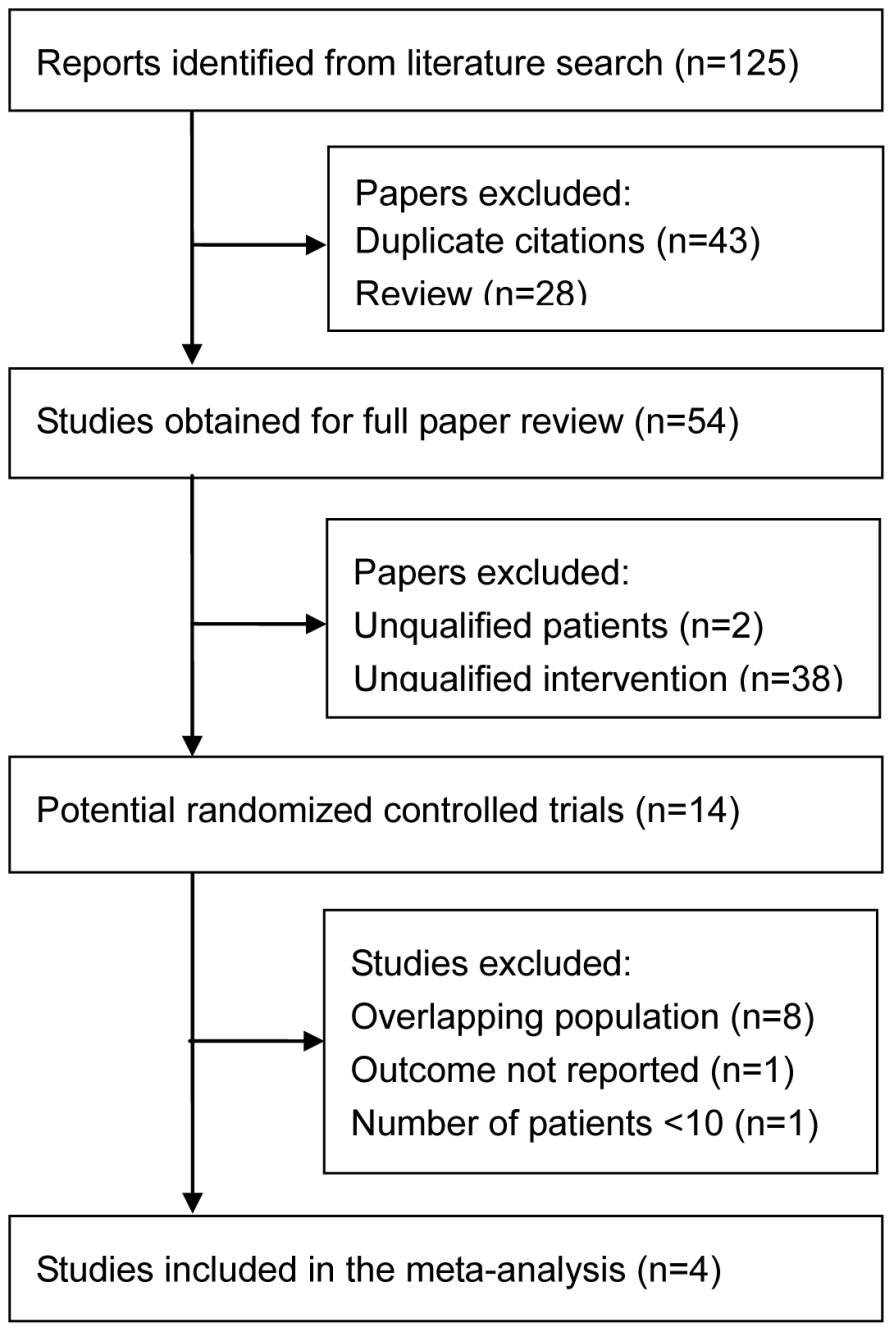
Flowchart of studies included in meta-analysis. RCT, randomized controlled trial.

The selection process and reasons for exclusion are detailed in Figure 1. The initial search identified 125 potentially relevant articles, of which 71 were excluded based on the titles and abstracts. The remaining 54 were retrieved for a full-text review, and 40 of them were excluded because 38 included unqualified patients, two involved unqualified interventions, eight contained duplicated data [22]–[29], one did not report outcomes of interest [30], and one contained only one patients (<10) in the ranibizumab arm [31]. Thus, four RCTs [13], [14], [32], [33] were included in the final meta-analysis.

### 2. Study Characteristics and Quality

The main characteristics of the four RCTs included in the meta-analysis are presented in Table 1, and the outcome data of each included trial are described in Table 2. These studies were published between 2012 and 2013. The sizes of the RCTs ranged from 317 to 1,185 patients (a total of 2,613; 1,291 with bevacizumab and 1,322 with ranibicizumab). Of the four trials, one was done in the USA [14], one in the UK [13], one in France [33], and one in Australia [32]. The trials included in this meta-analysis appeared to have been reasonably designed and conducted. All studies had a statement regarding randomization and double-blindness. Four trials described the methods of randomization. Four trials reported withdrawals and dropouts. All trials described the main outcome, and no missing data seemed to influence the results. The Jadad score of the studies included was 5.

**Table 1:** Baseline characteristics of the head-to-head studies comparing ranibizumab with bevacizumab.

Study	Location	Center	Blind	Duration	Intervention	No. of eyes	Age (years)	Male (%)	Visual acuity (letters)	Foveal thickness (mm)
CATT	USA	44	double	2 years	Ranibizumab Monthly	301	79.2±7.4	39.2%	60.1±14.3	458±184
					Bevacizumab Monthly	286	80.1±7.3	37.1%	60.2±13.1	463±196
					Ranibizumab as Needed	298	78.4±7.8	37.9%	61.5±13.2	458±193
					Bevacizumab as Needed	300	79.3±7.6	38.7%	60.4±13.4	461±175
IVAN	UK	23	double	2 years	Ranibizumab	314	77.8±7.6	41%	67·8±17·0	471.6±192.5
					Bevacizumab	296	77.7±7.3	39%	66·1±18·4	465.6±183.1
GEFAL	France	38	double	1 year	Ranibizumab	129	78.68±7.27	31.21%	55.78±13.99	354.75±109.90
					Bevacizumab	119	79.62±6.90	35.82%	54.62±14.07	359.21±120.72
MANTA	Austria	10	double	1 year	Ranibizumab	163	77.6±8.1	36.20%	56.4±13.5	365.0±8.1
					Bevacizumab	154	76.7±7.8	36.36%	57.0±13.0	374.6±8.4

CATT =  The Comparison of Age-related macular degeneration Treatments Trials; IVAN  =  The Alternative treatments to Inhibit VEGF in Age-related choroidal Neovascularization study; MANTA  =  The Multicenter Anti-VEGF Trial; GEFAL  =  The Groupe d'Etude Français Avastin versus Lucentis dans la DMLA néovasculaire (The French Study Group Avastin versus Lucentis for neovascular AMD).

**Table 2:** Outcome data of randomized controlled trials included in the meta-analysis.

Adverse events	CAAT		IVAN		GEFAL		MANTA	
	Ranibizumab (N = 599)	Bevacizumab (N = 586)	Ranibizumab (N = 314)	Bevacizumab (N = 296)	Ranibizumab (N = 246)	Bevacizumab (N = 255)	Ranibizumab (N = 163)	Bevacizumab (N = 154)
**Systemic adverse event**								
Death-all causes	32	36	15	15	3	2	2	3
Arteriothrombotic events	28^*^	29	13	10	1	1	3	5
Stroke	8	8	6	3	0	0	1	1
Nonfatal myocardial infarction	9	7	4	4	1	1	2	3
Vascular death	12	14	3	4	0	0	0	0
Venous thrombotic events	3	10	3	4	0	1	0	0
Hypertension	3	4	0	0	2	1	0	0
**MedDRA system organ class**								
Cardiac disorders	47	62	20	19	5	2	1	1
Infections	41	54	9	12	2	4	3	3
Nervous system disorders	34	36	9	8	0	3	1	2
Injury and procedural complications	23	35	12	10	2	4	3	2
Neoplasms benign and malignant	27	22	11	14	1	1	2	1
Surgical and medical procedures	0	0	16	14	0	5	0	1
Gastrointestinal disorders	11	28	3	9	5	3	0	0
Any other system organ class	81	104	25	27	11	10	2	3

### 3. Risk of Systemic Adverse Events

**Figure 2:**
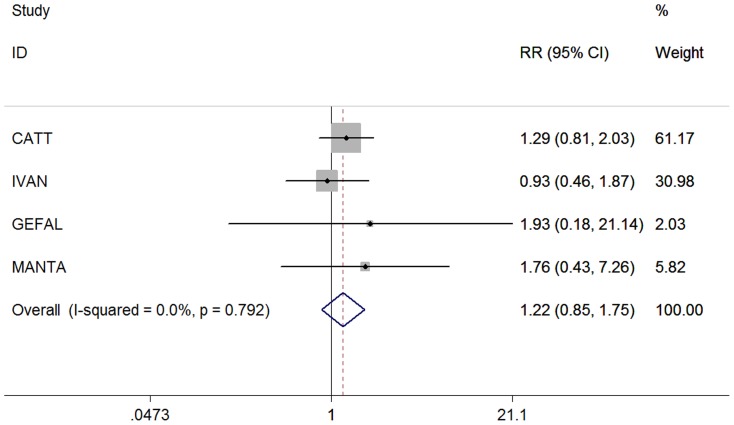
Risk ratio of thromboembolic events associated with intravitreal bevacizumab compared with ranibizumab. Each study is shown by the point estimate of relative risk “risk ratio” (RR) - the size of the square is proportional to the weight of the study - and 95%confidence interval for the RR (lines extending from the squares); the pooled RR and 95%confidence interval are shown as a diamond.

The risk estimates for systemic adverse events associated with intravitreal bevacizumab, as compared with ranibizumab, were summarized in Table 3. No significant differences between bevacizumab and ranizumab were found in terms of the incidence of death from all causes, arteriothrombotic events, stroke, nonfatal myocardial infarction, vascular death, venous thrombotic events, and hypertension, with the pooled RRs being 1.11 (0.77, 1.61), 1.03 (0.69,1.55), 0.84 (0.39,1.80), 0.97 (0.48, 1.96), 1.24 (0.63, 2.44), 2.38 (0.94, 6.04), and 1.02 (0.29, 3.62), respectively. When any arteriothrombotic or venous thrombotic events, such as TEE, were combined, no significant difference was detected (RR, 1.22; 95% CI: 0.85 to 1.75; P = 0.292) (Figure 2). Furthermore, when adverse events were divided by MedDRA system organ class, there was also no significant difference between bevacizumab and ranibizumab injections. The tests for heterogeneity were all non-significant (all P>0.1). We tested the robustness of our analyses by performing sensitivity analyses excluding the CATT study (largest trial). Excluding this study did not change our final results.

**Table 3:** Risk ratio of systemic adverse events associated with intravitreal bevacizumab compared with ranibizumab.

Avastin vs Lucentis	Study(n)	RR (95%CI)			Heterogeneity		Overall Effect	
		Estimate	Lower	Up	P	I^2^(%)	Z	P
Death-all causes	4	1.11	0.77	1.61	0.907	0.00%	0.56	0.572
Arteriothrombotic events	4	1.03	0.69	1.55	0.828	0.00%	0.15	0.879
Stroke	3	0.84	0.39	1.80	0.737	0.00%	0.46	0.649
Nonfatal myocardial infarction	4	0.97	0.48	1.96	0.925	0.00%	0.09	0.928
Vascular death	2	1.24	0.63	2.44	0.842	0.00%	0.61	0.541
Venous thrombotic events	3	2.38	0.94	6.04	0.676	0.00%	1.83	0.067
Hypertension	2	1.02	0.29	3.62	0.471	0.00%	0.03	0.977
MedDRA system organ class								
Cardiac disorders	4	1.20	0.88	1.62	0.460	0.00%	1.16	0.245
Infections	4	1.36	0.97	1.91	0.965	0.00%	1.79	0.074
Nervous system disorders	4	1.11	0.75	1.66	0.606	0.00%	0.52	0.602
Injury and procedural complications	4	1.31	0.87	1.98	0.578	0.00%	1.30	0.194
Neoplasms benign and malignant	4	0.96	0.62	1.48	0.743	0.00%	0.18	0.854
Surgical and medical procedures	3	1.75	0.43	7.16	0.222	33.60%	0.78	0.434
Gastrointestinal disorders	3	1.90	0.78	4.62	0.141	49.00%	1.41	0.158
Any other system organ class	4	1.25	0.99	1.56	0.803	0.00%	1.90	0.058

### 4. Publication Bias

Due to the limited number (<10) of studies included in each analysis, publication bias was not assessed.

## Discussion

The development of VEGF inhibitors has revolutionized the treatment of AMD. Bevacizumab and ranibizumab are the two most common VEGF inhibitors in ophthalmic practice [34]. Although anti-VEGF agents are injected into the eye in small quantities, concerns about systemic safety have been raised [35]. Until relatively recently, high-quality data comparing the efficacy and safety of ranibizumab and bevacizumab in AMD were lacking. Because many adverse events are relatively uncommon, clinical trials often lack the power to detect small but clinically important risk differences. Hence, meta-analyses pooling data from multiple studies provide important insights [15], [36]. The main aim of this study is to provide an evidence-based analysis of the safety profile for bevacizumab versus that of intravitreal ranibizumab injections in patients with AMD. In the present meta-analysis, we have reviewed the literature regarding the safety of intravitreal bevacizumab as compared with that of ranibizumab. The pooled results suggest that the incidence of specific systemic complications did not differ significant between bevacizumab and ranibizumab. Also, no heterogeneity was observed across the studies.

Several high-quality non-randomized studies [37]–[40] focusing on adverse effects for bevacizumab versus ranibizumab are summarized in Table 4. All of them reported that the rates of specific systemic adverse events, such as all-cause mortality, stroke, acute myocardial infarction, and venous thromboembolism during the bevacizumab and ranibizumab periods were not different. However, the limitation of these studies was that a non-randomized study design was used (case control or cohort study). The principal finding of our meta-analysis is consistent with the aforementioned studies on the topic.

**Table 4:** Summary of high-quality non-randomized studies comparing ranibizumab with bevacizumab.

Author, country	Design	Population	Method	Results
Campbell et al., 2012, Canada	Population based nested case-control study	Older adults with a history of physician diagnosed retinal disease identified between 1 April 2006 and 31 March 2011.	Cases were patients admitted to hospital for ischaemic stroke, acute myocardial infarction, venous thromboembolism, for congestive heart failure. Event-free controls were matched to cases on the basis of year of birth, sex, history of the outcome in the previous 5 years, and diabetes	Adjusted odds ratios for bevacizumab relative to ranibizumab were 1.03 (0.67 to 1.60) for ischaemic stroke, 1.23 (0.85 to 1.77) for acute myocardial infarction, 0.92 (0.51 to 1.69) for venous thromboembolism, and 1.35 (0.93 to 1.95) for congestive heart failure. Results showed these risks did not differ significantly between bevacizumab and ranibizumab injections.
Campbell et al., 2012, Canada	Population-based, time series analysis	All patients aged 66 years or older with physician-diagnosed retinal disease between 2002 and 2010 (N = 116 388).	Segmented regression analysis was used to evaluate changes in the rate of hospitalization for ischemic stroke associated with the introduction of bevacizumab and ranibizumab.	Bevacizumab trend change coefficient: -0.0026 stroke hospitalizations/1000 subjects/month (P = 0.20); Ranibizumab trend change coefficient: -0.0011 stroke hospitalizations/1000 subjects/month (P = 0.78). Results showed that stroke rates in the bevacizumab and ranibizumab periods were not different.
French et al., 2011, USA	Cohort study	Beneficiaries of the Veterans Health Administration aged ≥55 years with AMD in fiscal years 2007-2009 were included.	Anti-vascular endothelial growth factor exposure was identified through pharmacy records. Cox proportional hazard model was adjusted for age, gender, number of injections, and ocular and medical comorbidities.	The adjusted HR for all-cause mortality were 0.94 (95%CI: 0.72 to 1.22) for bevacizumab and 0.85 (95%CI: 0.67 to1.08) for ranibizumab. Results showed lack an association between the use of either ranibizumab or bevacizumab and mortality.
Curtis et al., 2010, USA	Cohort study	Medicare beneficiaries 65 years or older with a claim for AMD in fiscal years 2005-2006 were included.	When the patients received a therapy different from the initial therapy, the data were censored. The associations between anti-VEGF therapies and the risks of all-cause mortality, incident myocardial infarction, bleeding, and incident stroke were calculated.	Adjusted HRs for ranibizumab relative to bevacizumab were 0.90 (0.79-1.02) for all-cause mortality, 0.84 (0.66-1.06) for myocardial infarction, 1.03 (0.93-1.15) for bleeding, 0.81 (0.68-0.98) for stroke. Results showed these risks did not differ significantly between bevacizumab and ranibizumab injections.

Thus far, ranibizumab and bevacizumab have been evaluated in several systematic reviews [11], [17], [34], [41]. However, the published reviews focused on the beneficial effects and clinical effectiveness of VEGF inhibitors, without adequately addressing their adverse effects. Furthermore, they are mainly based on indirect comparative studies; this may lower the evidence level. In Schmucker and colleagues' report [41], only one multiple-center, head-to-head RCT (CATT) was included; it had relatively modest sample sizes. In another meta-analysis by Chakravarthy et al. [13], no difference in the frequency of death, arterial thrombotic events, or hospital admission for heart failure was recorded between the drugs. Their study is limited by the fact that only 1-year CATT data were included. In our study, we incorporated the 2-year CATT data and two other well-designed RCTs. We found a similar risk of specific adverse events between the bevacizumab and ranibizumab groups. From a theoretical viewpoint, the risk of the development of systemic adverse events may be higher with bevacizumab than with ranibizumab [10]. Bevacizumab is more likely to induce immunologic activation and will remain in systemic circulation than ranibizumab. Thus, bevacizumab administration may create a higher risk of systemic adverse events. These highlight the need for ongoing surveillance and large population-based studies to investigate these outcomes [15].

The results of this meta-analysis must be interpreted cautiously in light of the strengths and limitations of the included trials. A key strength of this study is the fact that all the studies included in this meta-analysis were published by established centers of excellence using a randomized controlled design and all of them were well-performed and of high quality. In addition, with the enlarged sample size, we have enhanced statistical power to provide more precise and reliable effect estimates. Despite our rigorous methodology, some limitations of the current study should not be ignored. First, we cannot fully exclude publication bias. The number of included studies is insufficient to carry out further statistical testing to detect publication bias through an asymmetry plot. In addition, we did not attempt to gain access to unpublished results. More RCTs are warranted to confirm or refute our finding in the future update meta-analysis. Second, all studies have come from western populations with predominately Caucasian participants. The relatively good distribution of the study population makes findings from this meta-analysis a fair representation of the general population. The safety of these drugs for other ethnicities, such as Asians, must be tested. Furthermore, patients enrolled in RCTs meet strict eligibility criteria, which may exclude many patients at a higher risk for systemic adverse events. These limitations likely resulted in an underestimation of the incidence of systemic adverse events. However, the determination of the risk of systemic adverse events associated with bevacizumab versus ranibizumab was not likely affected, because this underestimation should have had similar impacts on both arms. Finally, given that the treatment of wet AMD is not limited to 2 years, more data from studies of longer durations are needed to determine the relative safety of each anti-VEGF agent over the long term.

In conclusion, there is no difference between bevacizumab and ranibizumab in terms of the risk of specific systemic adverse events. However, the results should be interpreted cautiously because the relevant evidence remains limited, and the findings must be confirmed through future research involving well-designed cohort studies or RCTs.

## Supporting Information

Checklist S1 **PRISMA checklist.**
(DOC)
